# Human parvovirus B19 infection in malignant and benign tissue specimens of different head and neck anatomical subsites

**DOI:** 10.1186/s13027-023-00528-5

**Published:** 2023-09-14

**Authors:** Haniyeh Abuei, Sepide Namdari, Tahereh Pakdel, Fatemeh Pakdel, Azadeh Andishe-Tadbir, Abbas Behzad-Behbahani, Mohammad J. Ashraf, Parnian Alavi, Ali Farhadi

**Affiliations:** 1 Division of Medical Biotechnology, Department of Medical Laboratory Sciences, School of Paramedical Sciences, Shiraz University of Medical Sciences, Shiraz, Iran; 2 Diagnostic Laboratory Sciences and Technology Research Center, School of Paramedical Sciences, Shiraz University of Medical Sciences, Shiraz, Iran; 3 Department of Oral and Maxillofacial Pathology, School of Dentistry, Shiraz University of Medical Sciences, Shiraz, Iran; 4 Oral and Dental Disease Research Center, School of Dentistry, Shiraz University of Medical Sciences, Shiraz, Iran; 5 Department of Pathology, School of Medicine, Shiraz University of Medical Sciences, Shiraz, Iran; 6 Department of Medicine, University of Alberta, Edmonton, AB Canada

**Keywords:** Human parvovirus B19, HNSCC, Nested-PCR, IHC, HPV, p16INK4a, NF-κB

## Abstract

**Background:**

The role of human parvovirus B19 (B19V) infection in malignant and benign lesions such as head and neck squamous cell carcinomas (HNSCCs) and oral mucocele lesions has not been established. Herein, we examined, for the first time, the presence of B19V in HNSCCs from Iranian subjects.

**Methods:**

One hundred and eight HNSCC specimens were analyzed for the presence of B19V using nested polymerase chain reaction (nPCR) and TaqMan quantitative PCR assays. Immunohistochemistry procedures were performed to evaluate the expression of B19V VP1/VP2 proteins, p16INK4a, and NF-κB in tumor tissues and their adjacent non-tumor tissues. In addition, 40 oral mucocele, 30 oral buccal mucosa swabs, and 30 nasopharyngeal swabs obtained from healthy adults were analyzed as controls.

**Results:**

B19V DNA was detected in 36.1% of HNSCCs. Further, 23.3% of HNSCC specimens showed immunoreactivity against B19V VP1/VP2 proteins. There was a significant difference in the frequency of B19V DNA-positive cases between the patient and control groups (p < 0.0001). Moreover, comparing tumoral tissues and their adjacent non-tumor tissues in terms of immunoreactivity against B19V structural proteins, a significant association was found between tumor tissues and B19V infection (p < 0.0001). Finally, investigating the simultaneous presence of B19V and high-risk human papillomaviruses (HPV) DNA, we found a significant association between these two viral infections in HNSCCs (p = 0.031).

**Conclusions:**

To sum up, B19V was frequently present in HNSCC tissues of Iranian patients but mostly absent in the adjacent non-tumor tissues as well as oral mucocele lesions, buccal, and nasopharyngeal swabs of healthy subjects. HPV possibly contributes to B19V persistence in HNSCC tissues. Additional research is required to investigate potential etiological or cofactor roles of B19V in the development of HNSCCs.

## Background

Head and neck squamous cell carcinoma (HNSCC) refers to a group of cancers derived from the mucosal epithelium in the oral cavity, nasopharynx, oropharynx, hypopharynx, and larynx [1]. It has been reported to be the sixth most frequent cancer worldwide [2]. Some subsets of HNSCCs have been largely attributed to viral etiologies, namely oncogenic human papillomaviruses (HPV) and Epstein-Barr virus (EBV) [1]. In addition, other risk factors including tobacco and alcohol consumption and exposure to environmental pollutants have been reported for HNSCCs [1, 3]. However, the role of other viral agents such as parvovirus B19 (B19V) in HNSCCs has not been investigated extensively.

B19V is a widespread human pathogen infecting erythroid progenitor cells [4]. It is a non-enveloped single-stranded DNA virus with only two structural proteins, VP1 and VP2 [5]. B19V has been classified into three distinct genotypes (genotype 1, 2 and 3), with genotype 1 being the major variant circulating in the population worldwide [6]. The infection occurs through droplet nuclei and the respiratory route and may cause a variety of clinical manifestations [7]. Primary infection with B19V is often followed by the lifelong persistence of viral DNA in different human tissues such as tonsils, salivary glands, and thyroid [8].

Different reports have correlated the expression of B19V genes with alterations in the expression of cellular genes involved in inflammatory pathways such as nuclear factor kappa B (NF-κB) [9, 10]. B19V infection has also been associated with inflammatory changes in tumor microenvironment and may lead to tumor progression [10, 11]. However, despite several reports about the involvement of p16INK4a dysregulations in HNSCCs which occurs primarily due to viral etiologies such as HPV [12, 13], there exist no studies investigating its potential association with B19Vinfection in this group of carcinomas.

As a part of our research in assessing the role of viruses in human HNSCCs, we extended our HPV studies to investigate the presence of B19V DNA and B19V VP1/VP2 proteins and its relationship with p16INK4a and NF-κB expression in HNSCC specimens. To the extent of our knowledge, this study provides the first data on B19V infection in malignant and benign head and neck lesions.

## Methods

### Study subjects

One hundred and eight paraffin-embedded (FFPE) tissue specimens of primary HNSCC tumoral tissues which were histologically confirmed by two pathologists were included in this study. These samples were previously examined for the presence of high-risk HPV DNA as described elsewhere [14], and the data showed that 25 (23.1%) of the tumoral tissues were HPV-related HNSCCs. Furthermore, 40 histopathologically diagnosed FFPE oral mucocele specimens were collected as benign salivary gland lesions. All archived samples were obtained from department of oral & maxillofacial pathology, school of dentistry, Shiraz University of Medical Sciences. In addition, 30 oral buccal mucosa swabs and 30 nasopharyngeal swabs were obtained from healthy adults without apparent oral cavity lesions, infectious diseases, or inflammatory conditions. Moreover, volunteers with certain conditions such as pregnancy and immune deficiency (chemotherapy, immunosuppressive therapy or bone marrow transplantation) were excluded from the study. All participants gave written informed consent.

### DNA extraction and integrity analysis

For 108 HNSCC tumor samples, DNA was available from previous experiments. Genomic DNA was extracted from 8-µm serial sections of FFPE oral mucocele tissue specimens using QIAamp Tissue Kit (Qiagen, Hilden, Germany). Oral buccal and nasopharyngeal swabs were used to brush and collect cells from the subjects’ mucosa. DNA was isolated immediately after swabbing using the blackPREP Swab DNA Kit (Analytik Jena, Germany). Both extraction procedures were performed according to the manufacturers’ protocols. The adequacy of the DNA was measured using a UV-Vis spectrophotometer (WPA Lightwave II) and its quality was analyzed by ß-Globin gene PCR amplification using 500 ng of extracted DNA as described elsewhere [15].

### Detection of human parvovirus B19 DNA and sequencing

Two pairs of degenerate primers were designed using Allele ID version 7.5 (Premier Biosoft, California, USA) to detect all parvovirus B19 genotypes (Table 1). Using these primers, a 106-bp DNA fragment corresponding to the region between NS1 and VP1 viral genes was targeted. We confirmed that the primer pair would specifically amplify the sequence by searching for the nucleotide sequences that contain both primer sequences on opposing strands in the NCBI GenBank database using BLAST (http://www.ncbi.nlm.nih.gov/BLAST). A 406-bp synthetic DNA fragment (Bioneer, South Korea) derived from the unique region of the structural protein VP1 and a non-structural protein (NS1) of B19V cloned in pBHA vector was used as a template for the assay.

The first round of nested PCR was carried out in a 20-µL reaction mixture containing 13 µL Master Mix Red 2x (Ampliqon, Odense, Denmark), 500 ng of extracted DNA and a 0.7 µM of 4 A Forward and 4B Reverse outer primes. The reaction mixture for the second round of nested PCR was identical, except that 1 µl of the first reaction product and a 0.8 µM concentration of 4 C Forward and 4D Reverse inner primers were used. Reaction mixtures with the outer primer set were thermally cycled once at 94 °C for 3 min; 35 times at 94 °C for 1 min, 54 °C for 45 s, 72 °C for 45 s; and then once at 72 °C for 5 min. For the nested PCR products, reaction mixtures were thermally cycled once at 94 °C for 3 min, 40 times at 94 °C for 45 s, 56 °C for 45 s, and 72 °C for 45 s, and then once at 72 °C for 5 min. The 106-bp nested-PCR amplicons were detected in 1.5% agarose electrophoresis gels stained with SYBR Safe DNA gel stain (Thermo Fisher Scientific, USA) and visualized using a UV-transilluminator. In each round of amplification, plasmids containing B19V DNA and sterile water were included as positive and negative controls, respectively. The positive samples were analyzed in duplicate and the positive PCR products were submitted for automated DNA sequencing (Sequetech Corp., Mountain View, CA, USA) after purification using GF-1 PCR Clean-Up Kit (Vivantis, Malaysia) for further confirmation.

### Quantitative real-time PCR assay

Only the samples previously confirmed for harboring B19V DNA by nested-PCR assay were subjected to viral load quantification. For the in-house TaqMan quantitative PCR assay, the B19QF and B19QR primers, and B19Q TaqMan Probe (Table 1) were designed using Allele ID version 7.5 (Premier Biosoft, California, USA). The reaction mixture comprised 12.5 µl of RealQ Plus 2x Master Mix for Probe (Amplicon Odense M, Denmark), 0.4 µM of each primer, 0.18 µM of B19Q TaqMan Probe and 1–3 µl of each sample corresponding to 500 ng of extracted DNA in a final volume of 25 µL. The Rotor-Gene Q 5plex Platform real-time PCR system (QIAGEN, Germany) was used to provide the initial pre-incubation step at 95 °C for 15 min, followed by 40 PCR cycles at 94 °C for 20 s, 56 °C for 30 s, and 72 °C for 30 s each. In addition, all samples were subject to an in-house TaqMan qPCR assay for ß-Globin gene quantification in each experiment. The thermal cycler conditions and the reaction mixture for ß-Globin gene amplification were identical to B19V qPCR assay, except that 0.3 µM of each primer and 0.16 µM of ß-Globin TaqMan Probe (Table 1) were used [16].

**Table 1:** Sequences of the primers and probes used in this study

Primer name	Sequences (5′−3′)	Product size (bp)
4 A Forward (outer)	AACGCCTCAGAAAAATACCC	340
4B Reverse (outer)	TAAGTGCTGAAACTCTAAWGG	
4 C Forward (inner)	CAAAAGCATGTGGAGTGAGG	106
4D Reverse (inner)	CACYTTATAATGGTGCTCTGG	
B19QF	CCATATGACCCAGAGCACCA	83
B19QR	ACCTTTGCCTCCTTTCCACT	
B19Q Probe	FAM-CCCGCAGCAAGTAGCTGCCA-BHQ	
ß-Globin F	TGTGTTCACTAGCAACCTCAA	111
ß-Globin R	CTCACCACCAACTTCATCCA	
ß-Globin Probe	FAM-CCTGAGGAGAAGTCTGCCGTTACTGCC-BHQ	

W = A, T, Y = C, T, bp: base pair

### Standard curve

In order to generate a standard curve for ß-Globin gene quantitative analysis, a 111-bp amplified PCR product using ß-Globin F and ß-Globin R primers (Table 1) was cloned into pTZ57R/T vector using the InsTA- clone PCR Cloning Kit (Thermo scientific, USA). The artificially synthesized 406-bp B19V DNA fragment cloned in pBHA vector was also used to generate a standard curve for B19V DNA quantification. Both plasmids were transformed into *E. coli* DH5α competent cells and subsequently purified using GF-1 Plasmid DNA Extraction Kit (Vivantis Technologies, Malaysia). Based on the plasmid concentration measured by a NanoDropTM spectrophotometer (Nano DropTM2000; Thermo Scientific) and the size of each linearized plasmid, the absolute copy number of DNA in each sample was calculated. To generate the standard curve for cell quantification, ß-Globin gene-containing plasmids with 10-fold serial dilutions ranging from 6.7 × 10^8^ to 670 copies/reaction were used. For B19V genome quantification, a standard curve with tenfold serial dilutions ranging from 5.3 × 10^8^ to 530 copies/reaction in a background of 500 ng DNA extracted from FFPE HeLa cell block was generated. All the curves were computed based on the Ct values of the diluted standards using Rotor-Gene Q Series Software (QIAGEN, Germany/ version 2.0.2). Given that the number of cells in each sample can vary, leading to considerable change in viral load, B19V DNA copy number was normalized per 1000 cells. All the reactions were carried out in duplicate with the combination of B19V and ß-Globin gene amplification, enabling DNA copy number per cell estimation. Therefore, the absolute copy numbers of B19V DNA per cell were calculated as B19V DNA copy numbers/half of the total number of ß-Globin gene copies.

### Specificity and detection limit of qPCR assay

Using 100 ng of all available plasmids containing BKV, JCV, HPV-16, and HPV-18 complete viral genomes and 500 ng of the extracted DNA from previously confirmed clinical samples infected with HSV-1, EBV, CMV, HHV-6, and HHV-7, the specificity of B19V qPCR assay was determined. The detection limit of each test was determined based on the final dilution at which the florescent signal was amplified exponentially, using the PCR Ct value of ≤ 40 as a cut-off value.

### Human parvovirus B19, p16INK4a, and NF-κB immunohistochemistry

Immunohistological staining on 5-µm-thick FFPE tissue sections was performed using anti-B19V NCL-PARVO mouse monoclonal antibody (1:20 dilution, clone R92F6; Leica Biosystems, Novocastra, Newcastle upon Tyne, UK) for the identification of human B19V VP1/VP2 proteins. Moreover, mouse monoclonal antibodies anti-human p16INK4a (1:50; BD Pharmingen™; BD Biosciences, Franklin Lakes, NJ, USA) and anti-human NF-κB (p65) (1:400; Santa Cruz Biotechnology, Santa Cruz, CA, USA) were used for the examination of p16INK4a and NF-κB protein expression, respectively. After deparaffinization and rehydration, the sections were treated with 3% (v/v) hydrogen peroxide to block endogenous peroxidase activity. Heat-induced antigen retrieval was performed in 0.01 M sodium citrate (pH 6.0) and 10% (v/v) bovine serum albumin (BSA; Sigma, St Louis, MO, USA) in PBS at room temperature for 10 min to block the nonspecific antibody-binding sites. Slides were incubated with the monoclonal antibodies in a humidified chamber for 1 h at room temperature, followed by incubation with EnVision + Dual Link System-HRP solution (Dako, Glostrup, Denmark) for 30 min. 3, 30-Diaminobenzidine (DAB) was used as a chromogen in the presence of hydrogen peroxide. Slides were counterstained using Meyer’s Hematoxylin. Serial sections of tissues were also run in parallel with the placement of the primary antibody with PBS and mouse IgG1(AMS/Immunokontact, Abingdon, UK) as negative controls. Sections from FFPE placental tissue from a hydrops fetalis case previously tested positive for harboring B19V DNA and formalin-fixed HeLa cell block sections available from the previous study known positive for p16INK4a and NF-κB immunostaining were used as positive controls of the assays [17]. The cytoplasmic and nuclear expression of NF-κB p65 were determined separately to examine the NF-κB/relA signaling pathway in cancerous and non-cancerous cells. The interpretation of p16INK4a and NF-κB immunostaining findings was carried out as described elsewhere [18].

### Statistical analysis

Data were analyzed using SPSS version 21.0 (SPSS Institute, Chicago, IL, USA). Chi-square test or two-sided Fisher’s exact test were used to analyze the association of B19V PCR and/or B19V IHC results with anatomical sites, clinicopathologic characteristics, and the overexpression p16INK4a and NF-κB proteins, where appropriate. McNemar test was used to compare the expression of B19V VP1/VP2 protein in tumor tissues and the corresponding peritumoral tissues. Mann-Whitney U test and Kruskal-Wallis test were applied to compare mean viral copy numbers between two groups and more than two groups, respectively. Statistical significance was assumed at a P < 0.05 level.

## Results

### Demographic and histopathological features

The study included 63 male and 45 female HNSCC patients whose ages ranged from 20 to 88 years (mean 56 years, SD = 14.63 years) at the time of sampling. In addition, 17 men and 23 women between 25 and 47 years of age (mean = 33.7, SD = 7.43) with oral mucocele lesions and 28 male and 32 female healthy adults between 21 and 45 years of age (mean = 30.04, SD = 6.10) were included as control groups. Table 2 represents the histopathological data collected from medical records of patients with HNSCC. Data on tobacco smoking and alcohol use by the patients were not available for our study. All the samples included in the study had tested positive for ß-Globin gene PCR amplification.

**Table 2:** Associations between demographic/histopathological features and parvovirus B19 status among patients with head and neck squamous cell carcinoma

Characteristics		All Patients (%) N = 108	B19V DNA positive (%) N = 39	B19V DNA negative (%) N = 69	p-value	B19V IHC positive (%) ^†^N = 24	B19V IHC negative (%) ^†^N = 79	p-value
Age (years)	< 56	58 (53.7)	24 (61.5)	34 (49.3)	0.235	15 (62.5)	40 (50.6)	0.356
	≥ 56	50 (46.3)	15 (38.5)	35 (50.7)		9 (37.5)	39 (49.4)	
Gender	Male	63 (58.3)	25 (64.1)	38 (55.1)	0.419	15 (62.5)	45 (57)	0.647
	Female	45 (41.7)	14 (35.9)	31 (44.9)		9 (37.5)	34 (43)	
Anatomic site	Tongue	38 (35.2)	12 (30.8)	26 (37.7)	0.949	8 (33.3)	30 (38)	0.660
	Oral Cavity	34 (31.5)	13 (33.3)	21 (30.4)		7 (29.2)	25 (31.6)	
	Larynx	13 (12.0)	5 (12.8)	8 (11.6)		4 (16.7)	8 (10.1)	
	Hypopharynx	9 (8.3)	3 (7.7)	6 (8.7)		3 (12.5)	5 (6.3)	
	Nasopharynx	8 (7.4)	4 (10.3)	4 (5.8)		2 (8.3)	6 (7.6)	
	Tonsil	6 (5.6)	2 (5.1)	4 (5.8)		0	5 (6.3)	
Grade (Differentiation)	Well	61 (56.5)	26 (66.7)	35 (50.7)	0.191	13 (54.2)	44 (55.7)	0.746
	Moderately	33 (30.6)	8 (20.5)	25 (36.2)		6 (25)	26 (32.9)	
	Poorly	10 (9.3)	3 (7.7)	7 (10.1)		3 (12.5)	7 (8.9)	
	Not Recorded	4 (3.7)	2 (5.1)	2 (3)		2 (8.3)	2 (2.5)	
p16^INK4a^ Expression	Positive	32 (29.6)	13 (33.3)	19 (27.5)	0.498	6 (25)	26 (32.9)	0.610
	Negative	65 (60.2)	21 (53.8)	44 (63.8)		16 (66.7)	48 (60.8)	
	ND	11 (10.2)	5 (12.8)	6 (8.7)		2 (8.3)	5 (6.3)	
Cytoplasmic NF-κB Expression	High	73 (67.6)	27 (69.2)	46 (66.7)	0.816	18 (75)	55 (69.6)	0.599
	Low	28 (25.9)	9 (23.1)	19 (27.5)		5 (20.8)	23 (29.1)	
	ND	7 (6.5)	3 (7.7)	4 (5.8)		1 (4.2)	1 (1.3)	
Nuclear NF-κB Expression	High	24 (22.2)	9 (23.1)	15 (21.7)	0.812	7 (29.2)	17 (21.5)	0.411
	Low	77(71.3)	27 (69.2)	50 (72.5)		16 (66.7)	61 (77.2)	
	ND	7 (6.5)	3 (7.7)	4 (5.8)		1 (4.2)	1 (1.3)	

*IHC* immunohistochemistry, *NF-kB* nuclear factor kappa B, *ND* Not Determined

^†^Parvovirus B19 IHC staining was not performed on five specimens

### Nested PCR assay versus immunohistochemistry for the detection of parvovirus B19

When nested PCR assay was used to detect B19V DNA, it was revealed that thirty-nine (36.1%) HNSCC specimens harbored B19V DNA. BLAST analysis on the obtained sequencing data confirmed the presence of B19V DNA in 30 cases. The results of sequence analysis were inconclusive for the remaining nine samples. However, these samples were further confirmed to be infected with B19V by immunohistochemistry technique.

**Fig. 1:**
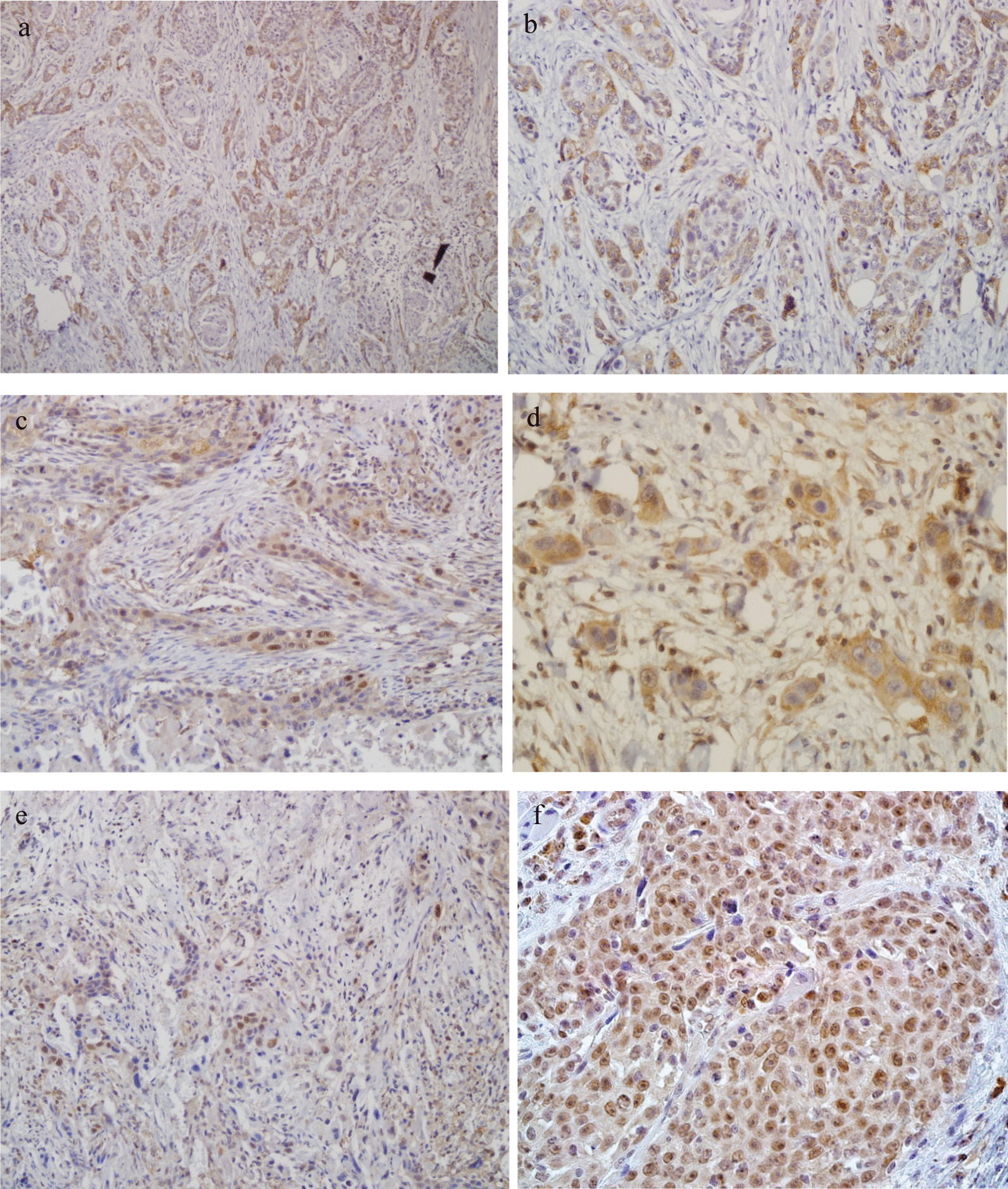
Immunohistochemistry staining of B19V VP1/VP2 antigens, NF-κB p65, and p16INK4a in moderately differentiated HNSCC with hematoxylin counterstain. **a**, **b** Represent positive B19V VP1/VP2 staining in the cytoplasm of many malignant HNSCC cells with 100× and 200× magnifications, respectively. **c**, **d** Represent nuclear and cytoplasmic NF-κB p65 immunostaining in HNSCC cells with the magnifications of 200× and 400×, respectively. **e**, **f** Indicate nuclear and cytoplasmic p16INK4a immunostaining in HNSCC cells with 200× and 400× magnifications

The immunohistochemical staining of B19V was carried out for 103 HNSCC cases (Fig. 1). Immunoreactivity against B19V VP1/VP2 proteins was detected in 24 (23.3%) cases, of which 22 (91.7%) cases had positive nested PCR results (p < 0.0001, Fisher’s exact test) and 5 (20.83%) had positive B19V VP1/VP2 immunoreactivity in their corresponding peritumoral tissues as well (p < 0.0001, McNemar test). Results of nested PCR in the control group revealed the presence of B19V DNA in one of 40 oral mucocele lesions (2.5%), four of 30 buccal swabs (13.3%), and two of 30 nasopharyngeal swabs (6.6%). IHC staining of B19V was also performed on 40 oral mucocele lesion specimens, none of which were found to be positive. There were no FFPE tissue specimens available for IHC analysis from other control groups who provided buccal or nasopharyngeal swabs for the study. Comparing the frequency of cases with positive B19V DNA in the control group with that in the patient group, we found a significant difference (p < 0.0001, Fisher’s exact test). Further, the difference remained significant when these controls were compared to HNSCC cases with immunoreactivity against B19V structural proteins. (p = 0.0015, Fisher’s exact test).

The data on the detection of B19V together with the previously available results of p16INK4a and NF-κB IHC assays were used to investigate potential associations between B19V infection and the overexpression of p16INK4a and NF-κB in HNSCC specimens. As presented in Table 2, B19V infection was revealed to be associated with neither p16INK4a nor NF-κB overexpression in these patients. Furthermore, associations between B19V infection and p16INK4a and NF-κB overexpression were examined in each anatomic site of HNSCC, individually (Table 3). No associations were found.

**Table 3:** p16^INK4a^ and NF-κB immunoreactivity in different anatomical sites of B19V-positive and -negative HNSCCs

		HNSCC anatomic site N (%)					
		Tongue N = 38	Oral cavity N = 34	Larynx N = 13	Hypopharynx N = 9	Nasopharynx N = 8	Tonsil N = 6
B19V DNA (+)	p16^INK4a^ (+)	3 (7.9)	4 (14.8)	2 (18.2)	0	2 (28.6)	2 (40)
	p16^INK4a^ (-)	9 (23.7)	5 (18.5)	3 (27.3)	3 (33.3)	1 (14.3)	0
B19V DNA (−)	p16^INK4a^ (+)	6 (15.8)	9 (33.3)	2 (18.2)	1 (11.1)	0	1 (20)
	p16^INK4a^ (-)	20 (52.6)	9 (33.3)	4 (36.4)	5 (55.6)	4 (57.1)	2 (40)
B19V DNA (+)	High cytoplasmic NF-κB expression	8 (21.6)	10 (31.3)	4 (33.3)	2 (25)	2 (28.6)	1 (20)
	Low cytoplasmic NF-κB expression	4 (10.8)	1 (3.1)	1 (8.3)	1 (12.5)	1 (14.3)	1 (20)
B19V DNA (−)	High cytoplasmic NF-κB expression	18 (48.6)	15 (46.9)	5 (41.7)	4 (50)	2 (28.6)	2 (40)
	Low cytoplasmic NF-κB expression	7 (18.9)	6 (18.8)	2 (16.7)	1 (12.5)	2 (28.6)	1 (20)
B19V DNA (+)	High nuclear NF-κB expression	0	4 (12.5)	3 (25)	2 (25)	0	0
	Low nuclear NF-κB expression	12 (32.4)	7 (21.9)	2 (16.7)	1 (12.5)	3 (42.9)	2 (40)
B19V DNA (−)	High nuclear NF-κB expression	5 (13.5)	4 (12.5)	1 (8.3)	3 (37.5)	1 (14.3)	1 (20)
	Low nuclear NF-κB expression	20 (54.1)	17 (53.1)	6 (50)	2 (25)	3 (42.9)	2 (40)

p16INK4a, cytoplasmic NF-κB, and nuclear NF-κB immunohistochemistry were successfully performed on 97, 101, and 101 of the total 108 HNSCC specimens, respectively

### Quantification of parvovirus B19 DNA

**Fig. 2:**
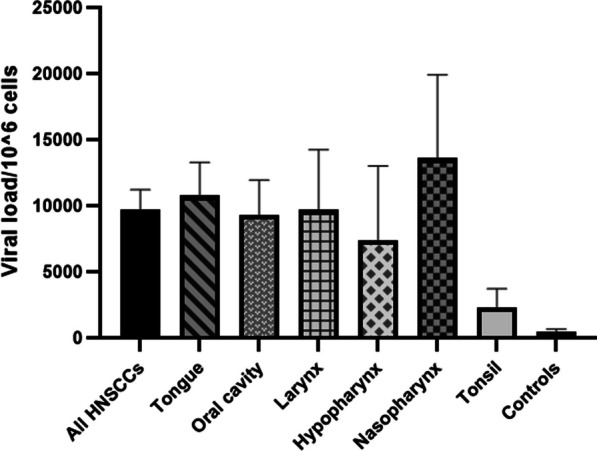
Mean B19 viral loads per 10^6^ cells in different anatomic sites of head and neck squamous cell carcinomas and control specimens

B19V DNA copy number was determined in all of 39 HNSCC cases and 7 controls with positive nested PCR results for B19V DNA, using real-time PCR assay and evaluated as copy number per 10^6^ cells using a standard curve. Viral copy numbers among HNSCC cases ranged from 940 to 26,260 copies/10^6^ cells and those in the control group ranged between 17 and 1680 copies/10^6^ cells. There existed a significant difference between the average number of B19V viral copies in HNSCC specimens of different anatomic sites and control specimens (p = 0.005, Kruskal-Wallis test) (Fig. 2). When analyzed individually using the Mann-Whitney U test, the average copy number of viral DNA in each anatomic subset of HNSCC was found to be significantly higher than the control group. Furthermore, the average viral copy number was significantly higher in patients with positive B19V IHC compared to those with negative IHC results (p = 0.007, Mann-Whitney U test). However, no associations were revealed between B19V viral load and histological grade, anatomic site of cancer, gender, age, and p16INK4a or NF-κB expression status.

### Co-infection of parvovirus B19 and HPV in HNSCC specimens

Sorted by anatomical site, B19V and high-risk HPV (16 and 18) coinfection was detected in 2 tonsil (33.3%), 2 larynx (15.4%), 2 nasopharynx (25%), 1 hypopharynx (11.1%), 4 oral cavity (11.8%), and 3 tongue (7.9%) SCC tumors. Examining the simultaneous presence of B19V and HPV DNA revealed a significant association between these two viral infections in HNSCC cases (p = 0.031, Fisher’s exact test).

## Discussion

To date, we are the first group to detect B19V in HNSCC tissue using molecular and IHC assays. In this study, we found that B19V was frequently present in HNSCCs in Iranian patients. Our data report the occurrence of B19V infection in 37.9% of HNSCC specimens. In contrast, the presence of B19V was only detected in 2.5%, 13.3% and 6.6% of oral mucocele lesions, buccal, and nasopharyngeal swabs of healthy subjects, respectively. A study by Dickinson et al. reported the presence of B19V DNA in 7 out of 10 HNSCC patients, 4 of whom had B19V DNA in both cancerous and healthy tissues while the remaining three only had viral DNA in their healthy tissues [19]. However, the significance of B19V DNA present in the healthy tissues of HNSCC patients remained unclear. Despite its strong tropism for cells of the erythroid lineage and its significant association with the related acute diseases, B19V has been detected in up to 50% of biopsy specimens from multiple non-erythroid tissues including brain, bone marrow, heart, liver, kidney, lung, thyroid, colon, testis, skin, synovium, tonsil, and lymphoid tissue persisting for decades after infection [8]. Multiple assays have been employed to detect B19V DNA in tissues with persistent B19V infection including in situ hybridization, Southern blotting, and touchdown, nested, and real-time PCR, among which, the sensitivity of nested PCR assay has been suggested to be the highest, detecting one genome per microliter of sample [20]. Using this method, B19V DNA can be detected even in a small number of cells of the tissue mass. There have been studies reporting immunohistochemical detection of B19V capsid proteins in liver, heart, colon, skin, cardiac interstitial cells, thyroid epithelial cells, synovial cells, mononuclear cells, and the seminiferous tubules of the testes [11, 21–26], suggesting the possible presence of intact viral particles in persistently infected tissues. Pyöriä et al. have demonstrated that long-lived lymphoid cells in the connective tissue of the tonsil account for lifelong B19V DNA tissue persistence [27]. Further, other studies have reported the presence of B19V capsid proteins in lymphoid cells of inflamed synovial tissues [28, 29]. However, the exact mechanism and significance of B19V persistence in the non-erythroid tissues are yet to be discovered. Taking into account all these data along with those from our study, it can be concluded that B19V is also likely to persist in head and neck tissues. Moreover, our study demonstrates that B19V preferentially persists in HNSCC tumoral tissues rather than their adjacent non-tumor tissues.

In the current study, a significant difference was observed in the frequency and average number of B19V viral copies between HNSCC and control specimens. However, the presence of B19V DNA, the immunoreactivity of viral VP1/VP2 proteins, and B19V viral load were not associated with histological grade and the anatomic site of HNSCC. For instance, B19V DNA and the expression of VP1/VP2 proteins were detected in 21.3% and 42.62% of well-differentiated, 18.18% and 24.24% of moderately-differentiated, and 30% and 30% of poorly-differentiated HNSCCs. This finding implies that the presence of the virus may not indicate a causative role in the development and progression of HNSCC but rather an opportunistic viral infection that thrives in the immunodeficient microenvironment of the tumor. Furthermore, the DNA replication of parvoviruses is S phase-dependent. B19V is capable of inducing cell cycle arrest at late S phase which facilitates the use of host factors for its DNA replication [30]. However, some parvovirus species including canine parvovirus lack the S phase induction mechanisms. Therefore, these viruses can only replicate in proliferating cells which is the reason for their tropism to tumor cells [31]. Accordingly, it is feasible to conclude that while most of the non-erythroid healthy cells including the studied normal oral mucosa and nasopharyngeal epithelial cells are quite resistant to B19V infection, they become sensitive following their transformation. However, since there have been no studies to date indicating progeny virion production of B19V in HNSCC cells in vitro, it is possible that B19V enters these cells, only completes a single step of double-stranded DNA conversion, and expresses VP and NS1 proteins in the host cells. To sum up, B19V possibly enters and persists in different non-erythroid cells, but the infection has not been proved to be productive or to cause disease.

There is strong evidence that high-risk HPV infection plays a major causative role in a subset of HNSCCs [32]. In the last few decades, there has been a decrease in the incidence of some HNSCCs in western countries due to lifestyle changes such as less smoking. However, the rate of oropharyngeal squamous cell carcinoma (OPSCC) has been rising in association with HPV. On the other hand, many patients with high-risk HPV infection never develop SCCs. One possibility is that co-infection with other viral agents plays a part as a co-factor in the development of SCCs [33]. A recent study by Poluschkin et al. reported a significant association between HPV DNA positivity and polyomavirus JC infection in HNSCC specimens [34]. In our study, 14 out of 39 B19V-infected HNSCC tissue specimens were positive for HPV DNA. The presence of B19V DNA in this study was statistically associated with HPV infection. There exists no previous study on B19V and HPV co-infections in HNSCCs. However, apart from the independent carcinogenic potential of HPV, an auxiliary oncogenic role of B19V when combined with HPV infection deserves consideration. It is nevertheless more likely that alterations in the expression of particular cellular factors caused by HPV co-infection contribute to the expression of B19V capsid protein in some tumor microenvironments. While in many cancer cells, the activation of wild-type p53 results in p53-mediated apoptosis, it is well-documented that the majority of HNSCC tumors, including HPV-negative ones, have TP53 mutations [35]. Given the fact that B19V induces apoptosis in order to release mature virions from the infected cells through the broken nuclear membrane [6], and considering several pieces of evidence suggesting that p53 is involved in the apoptotic pathway in B19-infected cells [36], it can be concluded that the persistence of B19V infection in HNSCC tumors may be considered as a direct result of TP53 mutation, a phenomenon that has already been reported in B19-positive bone marrow giant proerythroblasts [37] and anaplastic thyroid carcinoma tumors [38]. Moreover, in HPV-positive HNSCCs, the expression of HPV E6 protein leads to the disruption of p53 functions by targeting it for proteasomal degradation [39], which may contribute to B19V persistence in these tumor tissues.

Finally, further studies are required to find out whether B19V constantly replicates and expresses its proteins. Furthermore, given the persistence of B19V in various tissues including different anatomical sites of head and neck region, further research should be carried out to discover the exact mechanisms by which B19V enters these cells.

## Conclusions

Human parvovirus B19 is frequently present in HNSCCs from Iranian patients and is almost absent in oral mucocele lesions, buccal, and nasopharyngeal swabs of healthy subjects. In addition, B19V coexists in a subset of HPV-associated HNSCC tumors. Further studies are required to investigate a potential etiological or cofactor role of this virus in the development and/or progression of HNSCCs.

## Data Availability

This study is original and reflects the authors’ own research and analysis in a truthful and complete manner. Data are available upon request.

## Abbreviations

B19V: Human parvovirus B19
EBV: Epstein-Barr virus
FFPE: Paraffin-embedded
HNSCC: Head and neck squamous cell carcinoma
HPV: Human papillomaviruses
IHC: Immunohistochemistry
NF-κB: Nuclear factor kappa B
nPCR: Nested polymerase chain reaction
